# Proteomic analysis identifies highly expressed plasma membrane proteins for detection and therapeutic targeting of specific breast cancer subtypes

**DOI:** 10.1186/s12014-018-9206-0

**Published:** 2018-09-19

**Authors:** Yvonne S. Ziegler, James J. Moresco, Patricia G. Tu, John R. Yates, Ann M. Nardulli

**Affiliations:** 1 Department of Molecular and Integrative Physiology, University of Illinois at Urbana-Champaign, Urbana, IL USA; 2 Department of Molecular Medicine, The Scripps Research Institute, La Jolla, CA USA

**Keywords:** Individualized medicine, Plasma membrane proteins, Proteomic analysis, Estrogen receptor α-positive breast cancer, HER2-positive breast cancer, Triple negative breast cancer, Diagnostic markers

## Abstract

In recent years, there has been an emphasis on personalizing breast cancer treatment in order to avoid the debilitating side effects caused by broad-spectrum chemotherapeutic drug treatment. Development of personalized medicine requires the identification of proteins that are expressed by individual tumors. Herein, we reveal the identity of plasma membrane proteins that are overexpressed in estrogen receptor α-positive, HER2-positive, and triple negative breast cancer cells. The proteins we identified are involved in maintaining protein structure, intracellular homeostasis, and cellular architecture; enhancing cell proliferation and invasion; and influencing cell migration. These proteins may be useful for breast cancer detection and/or treatment.

**Electronic supplementary material:**

The online version of this article (10.1186/s12014-018-9206-0) contains supplementary material, which is available to authorized users.

## Introduction

Much has been written about the promise of personalized medicine for cancer treatment. Tremendous strides could be made in cancer treatment if targeted therapies were developed for specific tumor subtypes rather than relying on broad-spectrum chemotherapeutic agents. To begin to meet this challenge, it will be necessary to identify proteins that are expressed in individual patient tumors so that targeted treatments can be developed. Some progress has been made in this regard in the identification of proteins that are expressed in different classes of breast cancer (BC) tumors [1, 2].

The majority of BC tumors express estrogen receptor α (ERα) and the progesterone receptor (PR). Estrogen receptor antagonists and aromatase inhibitors have been successfully utilized to treat these ERα-positive tumors [3]. Another class of BC tumors do not express ERα, but overexpress the plasma membrane (PM) protein kinase HER2 (receptor tyrosine-protein kinase HER2). Humanized antibodies that bind to HER2 and reduce proliferation of HER2-positive BC cells have been utilized as a targeted treatment for this class of BC tumor [4]. Unfortunately, there exists a class of BC tumors for which no targeted treatments exist. These BC tumors do not express ERα, PR, or HER2 and are referred to as triple negative breast cancer (TNBC) tumors. There is a pressing need to identify proteins that are expressed by individual BC tumors, especially TNBC tumors so that new, targeted treatments might be developed.

PM proteins make attractive therapeutic targets due to their accessibility and involvement with the initiation of critical cell signaling cascades [5]. As proof of concept, many targeted therapies approved or in development target cell surface proteins [6]. Importantly, novel immunotherapies can capitalize on abundant cell-surface markers that are specific for particular cancer subtypes [7, 8].

The proteins discussed herein are an addendum to a previous study [6] in an attempt to identify additional proteins that might be useful targets for personalized therapy. The earlier analysis examined overexpressed proteins in several categories, including tyrosine kinases, MHC class I proteins, cell adhesion proteins, GPCRs and G proteins, cytoskeletal proteins, intermediate filaments, tubulins, actins, and myosins. The proteins discussed below did not readily fall into these functional categories and are now being considered based on their expression across the clinical classes of breast cancer.

## Materials and methods

### Cell lines and culture

MCF-7, MDA-MB-231, and SK-BR-3 cells, derived from pleural effusions (metastatic sites), and MCF-10A cells, derived from a benign fibrocystic mammary lesion, were originally obtained from ATCC (Manassas, VA) and maintained as described [6]. Two TNBC cell lines, DT22 (basal claudin-low) and DT28 (basal-epithelial) were derived from dissociated primary tumors and maintained as described [6, 9].

### Plasma membrane isolation

Purified PM were prepared using differential centrifugation followed by aqueous two-phase partitioning [6, 10, 11]. Briefly, ≥ 4 × 10^7^ cells were harvested and pelleted in PBS at 4 °C, resuspended (10^8^ cells/ml) in hypotonic buffer (0.2 mM EDTA, 1 mM NaHCO_3_ with protease inhibitors), and the nuclei and intact cells were removed by low-speed centrifugation (10 min at 800×*g*). The supernatant was subjected to high-speed centrifugation (1 h at 100,000×*g*) to yield a crude membrane pellet. The pellet was resuspended in 200 mM phosphate buffer, pH 7.2 and combined with the two-phase solution comprised of 6.6% dextran T500 (Sigma, St. Louis, MO) and 6.6% w/w polyethylene glycol (Emerald Bio, Bainbridge Island, WA) in 200 mM phosphate buffer, pH 7.2, vigorously mixed, spun (5 min at 1150×*g*), and the top phase containing PM was removed. The bottom phase was re-extracted with fresh dextran/polyethylene glycol buffer and combined with the first top phase. Finally, the pooled top phase was diluted with 5 volumes of 1 mM NaHCO_3_ and spun (1 h at 100,000×*g*). The PM pellets were flash frozen and stored at − 80 °C for subsequent MS analysis.

### Mass spectrometry

Pellets from ultracentrifugation were resuspended in 1 ml extraction buffer (635626, Clontech, Mountain View, CA,), followed by precipitation of 100 μg of protein in 23% TCA. Acetone-washed pellets were resuspended in 60 μl digestion buffer (0.1% Rapigest (Waters, Milford, MA) plus 50 mM ammonium bicarbonate) and heated at 60 °C for 30 min. Proteins were reduced with 5 mM tris(2-carboxyethyl)phosphine hydrochloride (C4706, Sigma) and alkylated with 10 mM iodoacetamide (Sigma). Proteins were then digested for 18 h at 37 °C in 1 μg trypsin (V5111, Promega, Madison, WI). Digestion was terminated by addition of 5% formic acid followed by a 30 min incubation at 37 °C. Debris was removed by centrifugation, 30 min at 18,000×*g*. MudPIT analysis was performed using an Accela HPLC pump (Thermo) and LTQ XL (Thermo) using an in-house built electrospray stage [12]. Protein and peptide identification were done with Integrated Proteomics Pipeline—IP2 (Integrated Proteomics Applications, San Diego, CA. http://www.integratedproteomics.com/). The tandem mass spectra were searched against a human protein database (UniprotKB, release 2018_07) with reversed sequences using ProLuCID (version 1.3.5) [13–15]. The search space included half and fully tryptic peptide candidates that fell within the mass tolerance window with no miscleavage constraint. Carbamidomethylation (+57.02146 Da) of cysteine was considered as a static modification. Data was filtered using DTASelect v2.0.49 with a protein false positive rate of 1%.

### TCGA analysis

TCGA data was extracted and analyzed as described [16]. Briefly, level 3 gene expression (microarray) data derived from breast invasive carcinoma were downloaded from the TCGA Research Network (http://cancergenome.nih.gov/) and parsed with a script generated at the University of Illinois Life Sciences Office of Information Technology. Data from 321 estrogen receptor positive, 53 HER2 positive, and 80 TNBC tumors were used to generate boxplots in GraphPad Prism (version 5.00 for Windows, GraphPad Software, La Jolla California USA, www.graphpad.com).

## Results and discussion

To identify proteins expressed by specific classes of BC tumors, we utilized MCF-7 cells, (ERα and PR positive), SK-BR-3 cells (HER2 overexpression), and MDA-MB-231 cells, which do not express ERα, PR, or HER2 and are classified as TNBC cells. Each of these cell lines has been extensively characterized and their gene expression profiles are similar to their respective tumor subtypes [17, 18]. In addition, two more recently isolated TNBC cell lines, DT22 and DT28 cells [6, 9, 16] were included to more carefully characterize the behavior of primary TNBC tumors. Using microarray analysis, the PAM50 classifier [19], and the claudin-low predictor [20], DT22 is classified as basal-claudin-low, and DT28 is classified as basal [9]. MCF-10A cells were included as a benign control [21, 22].

To exploit the fact that PM proteins make attractive therapeutic targets, PM proteins were isolated from each cell line and mass spectrometry (MS) analysis was used to define the PM proteomes and identify potential targets [6]. Data was parsed using spectral counts (SC), normalized spectral abundance factor (NSAF) [23], which corrects for the effect of protein length on spectral counts, and exponentially modified protein abundance index (EMPAI) [24], which is useful in quantifying protein content in complex mixtures (Additional file 1: Table S1). Biological validation of the MS data was previously performed using RT-PCR, immunofluorescence, and Western blotting analyses, and a close correlation was observed between the SC values and mRNA and protein levels in each of the cell lines [6]. Thus, the MS data generated is semi-quantitative in nature and can yield insights into differences and similarities among the BC and control cell lines.

Although a large number of proteins with higher PM expression have been previously described [6], this paper describes additional proteins that may be useful in targeted BC treatment or tracking disease progression and/or recurrence. Two factors were considered in selecting these proteins for further investigation, including the total number of SCs detected and also the number of SCs detected in a BC cell line compared to the MCF-10A control. Four BC PM proteins that have been successfully targeted or are in clinical trials as potential targets were examined in our data, including HER1 (EGFR), HER2 (ERBB2), HER3 (ERBB3), and the tyrosine-protein kinase receptor UFO (AXL) (Table 1). We decided upon a cut-off of 100 SCs for consideration of a protein, a value well above the 53 and 58 SCs seen for ERBB3 and AXL, respectively. Since targetability requires that the malignant cells stand out from normal cells, we also examined the ratio of SCs between the BC cells and control MCF-10A cells. Epidermal growth factor receptor (EGFR) provided the cut-off value of 8, thus requiring that malignant expression be at least eightfold higher than control expression to be considered.

**Table 1:** Targeted therapies approved or in clinical trials

Therapeutics and their targets							
Therapeutic	Gene target	MCF-7	SKBR3	DT22	DT28	MB-231	MCF-10A
Herceptin	*ERBB2*	0	276	45	3	3	0
Gefitinib, Tarceva, Poziotinib	*EGFR*	0	22	11	27	88	10
MM-111, Seribantumab, U3-1402	*ERBB3*	3	6	52	3	0	0
Bemcentinib	*AXL*	0	0	0	0	58	0

The therapeutic, gene target, and number of SCs identified in each cell line for that target are listed. MB-231 indicates MDA-MB-231 cells

### ERα-positive cells

**Fig. 1:**
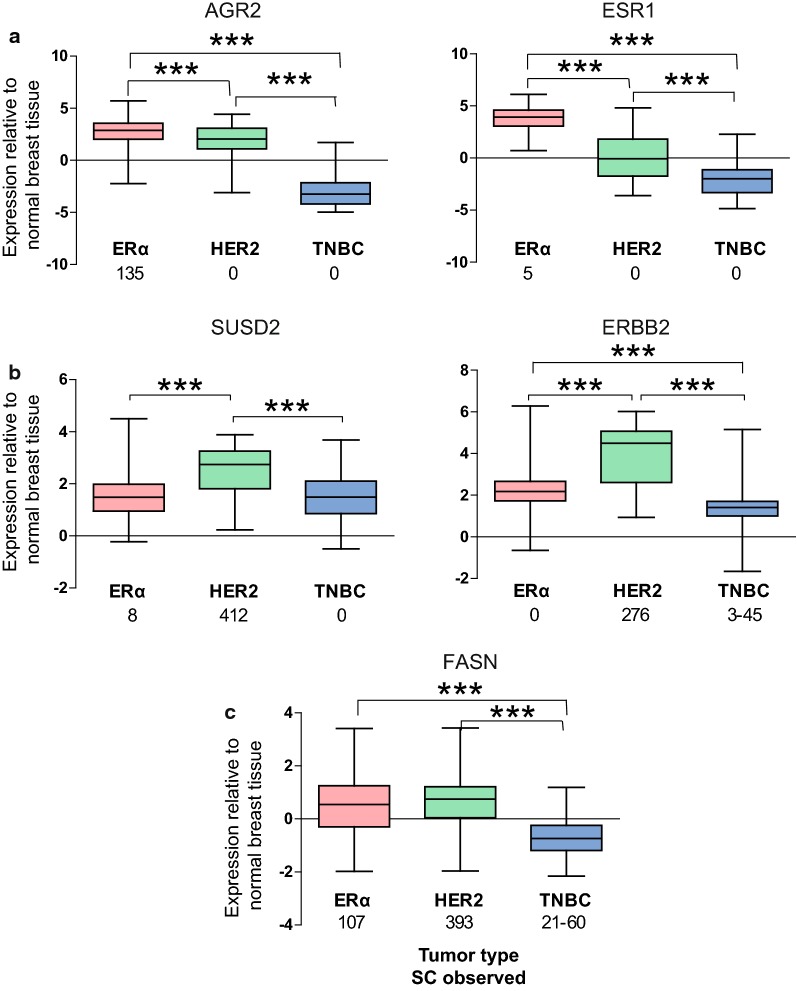
Gene expression in human BC tumors. Gene expression in ERα-positive BC, HER2-positive BC, and TNBC tumors are indicated in boxplots. Values are relative to normal breast tissue, with log2 Lowess normalized value > 0 indicating expression higher than and < 0 indicating expression lower than in normal tissue. Data are derived from The Cancer Genome Atlas (TCGA, https://cancergenome.nih.gov/). Indicated below each tumor subtype is the number of SCs identified by our proteomic analyses. Because TNBC SCs are derived from 3 cell lines, these values are sometimes presented as a range (panels **b** and **c**). ERα and HER2 (panels **a** and **b**, respectively) are provided for comparison. ***p < .001

AGR2 (anterior gradient protein 2 homolog) was highly expressed in MCF-7 cells with 135 SCs (Table 2), but was not detected in the PM of any of the other BC cell lines. AGR2 is a member of the protein disulfide isomerase (PDI) family, is involved in protein folding and disulfide exchange reactions, and has been implicated in cancer cell proliferation and progression [25, 26]. There is evidence to suggest that ERα induces AGR2 expression [27] and that higher AGR2 levels are associated with tamoxifen resistance in advanced disease [28]. To investigate how our MS results relate to clinical data, the gene expression of AGR2 was analyzed using microarray data from The Cancer Genome Atlas (TCGA). Consistent with our findings in MCF-7 cells, the expression of AGR2 is higher in ERα-positive BC tumors than in HER2-positive or TNBC tumors (Fig. 1a). Gene expression of ERα in BC tumors is included for comparison, but note that ERα is predominantly a nuclear protein with minimal PM expression, as reflected in our data [29]. Interestingly, we previously demonstrated that another protein in the PDI family (P4HB) alters ERα structure and influences estrogen-regulated gene expression [30]. AGR2, which enhances BC cell proliferation and has been detected in circulating tumor cells, may be useful as a marker of metastasis [26].

**Table 2:** Highly expressed plasma membrane proteins in breast cancer cell lines

Overexpressed proteins identified							
Gene	MCF-7	SK-BR-3	DT22	DT28	MB-231	MCF-10A	Protein
ERα-positive cells							
*AGR2*	***135***	0	0	0	0	0	Anterior gradient protein 2 homolog
*GCN1*	***107***	37	81	52	32	7	eIF2-alpha kinase activator GCN1
HER2-positive cells							
*ACLY*	57	***199***	73	83	78	19	ATP-citrate synthase
*DHRS2*	0	***200***	0	0	0	0	Dehydrogenase/reductase SDR family member 2
*SUSD2*	8	***412***	0	0	0	4	Sushi domain-containing protein 2
TNBC							
*EEF2*	85	57	***103***	52	75	9	Elongation factor 2
*TNC*	0	0	***105***	6	0	0	Tenascin
*PSMA5*	25	11	51	***219***	33	6	Proteasome subunit alpha type-5
*BASP1*	0	2	0	0	***140***	2	Brain acid soluble protein 1
*SLC1A5*	18	10	34	14	***115***	6	Neutral amino acid transporter B(0)
*ECPAS*	59	71	***109***	***118***	44	6	Proteasome adapter and scaffold protein ECM29
*TCP1*	16	66	***106***	***109***	74	8	T-complex protein 1 subunit alpha
ERα-positive and HER2-positive cells							
*FASN*	***107***	***393***	46	21	60	30	Fatty acid synthase
ERα-positive and TNBC cells							
*PA2G4*	***129***	84	54	***130***	26	15	Proliferation-associated protein 2G4
HER2-positive and TNBC cells							
*AHNAK*	91	***426***	***931***	142	***1219***	103	Neuroblast differentiation-associated protein AHNAK
*TPP2*	45	***119***	***150***	41	58	6	Tripeptidyl-peptidase 2
Multiple cell types							
*EEF1A1*	***894***	***834***	***576***	***486***	***560***	34	Elongation factor 1-alpha 1
*EEF1A2*	***739***	***525***	***157***	87	***154***	11	Elongation factor 1-alpha 2

The gene name, number of spectral counts identified in each cell line, and protein name are listed. SCs of more highly expressed proteins are bolditalic. MB-231 indicates MDA-MB-231 cells

GCN1 (eIF2-alpha kinase activator GCN1) is most highly expressed in MCF-7 cells (107 SCs) and has very low expression in MCF-10A cells (7 SCs). This protein activates the protein kinase GCN2 which orchestrates the cell’s response to amino acid starvation [31].

### HER2-positive cells

Three proteins were more highly expressed in the HER2-positive SK-BR-3 cells than in the other BC cell lines. ACLY (ATP-citrate synthase) was highly expressed in SK-BR-3 cells (199 SCs) and is responsible for acetyl-CoA production and de novo lipid synthesis. ACLY has been implicated in promoting high rates of aerobic glycolysis and lipid synthesis in a variety of tumor cells and in increasing tumor cell aggression [32]. Because reduction of ACLY expression leads to arrest of tumor cell proliferation in vitro and in vivo, it has been suggested that this protein may be useful as a therapeutic target [33]. DHRS2 (dehydrogenase/reductase SDR family member 2) metabolizes a variety of steroids and other cellular components and was highly and exclusively expressed in SK-BR-3 cells (200 SCs). Increased DHRS2 expression has also been noted in prostate [34] and ovarian [35] cancer cells. SUSD2 (sushi domain-containing protein 2) is involved in cell adhesion and migration and was highly expressed in SK-BR-3 cells (412 SCs). Interestingly, SUSD2 expression closely resembles the expression of HER2 in BC tumors (Fig. 1b). Elevated expression of SUSD2 is associated with increased tumor invasion and decreased survival in a mouse model [36]. Silencing of SUSD2 expression in endometrial carcinoma cells results in cell death [37]. Thus, it has been suggested that reducing expression of this protein might be a therapeutically useful strategy. In contrast to its effect on BC and endometrial cells, SUSD2 may function as a tumor suppressor in both kidney and lung cancer cells [38].

### TNBC cells

There is a tremendous need to identify proteins that are overexpressed in TNBC tumors in order to develop targeted treatments for these patients. We identified 7 proteins that are most highly represented in TNBC cells. DT22 cells overexpressed two of these proteins. EEF2 (elongation factor 2; 103 SCs) is overexpressed in multiple cancer types and is a promising immunotherapy target [39]. TNC (tenascin; 105 SCs), an extracellular matrix protein involved in neuronal guidance and axonal outgrowth in the brain, has been implicated in the metastasis of breast cancer to the lungs [40]. PSMA5 (proteasome subunit alpha type-5) is highly expressed only in DT28 cells (219 SCs). Increased proteasome activity has been noted in breast and other cancer types, providing protection from apoptosis, thereby yielding additional avenues for drug intervention [41].

Like the DT22 cells, MDA-MB-231 cells overexpressed two proteins, including BASP1 (brain acid soluble protein 1; 140 SCs). We were intrigued by the fact that, like TNC, BASP1 is involved in neuronal guidance in the brain [42, 43]. In addition, BASP1 is involved in terminal end bud formation during mammary gland development [44]. It seems possible that these two proteins may also play a role in tumor cell infiltration. SLC1A5 (neutral amino acid transporter B(0); 115 SCs), mediates uptake of glutamine in TNBC [45]. SLC proteins by definition are membrane transport proteins. Interestingly, another SLC protein, SLC39A6 is a PAM50 gene [46] and is most likely a Zn transporter [25]. The modified and excessive nutritional needs of cancer cells likely require increased expression of multiple SLC proteins.

Two proteins were more highly expressed in the TNBC cell lines derived from primary tumors, DT22 and DT28. The first of these, ECPAS (proteasome adapter and scaffold protein ECM29; 109 and 118 SCs, respectively), is a component of the 26S proteasome. Lower levels of ECM29 predict a better outcome in breast cancer patients. In fact, the therapeutic action of palbociclib involves activation of the proteasome through the loss of ECM29, resulting in cell senescence and decreased cell proliferation [47]. Also more highly expressed by DT22 and DT28 cells is TCP1 (T-complex protein 1 subunit alpha; 106 and 109 SCs respectively). This protein is part of the protein folding CCT chaperone complex that has generated interest as a therapeutic target in small cell lung cancer [48].

The fact that these seven proteins differentially distribute over the three different TNBC cell lines and that 5 of the proteins were overexpressed by just one TNBC cell line reflect that the TNBC category is heterogeneous and requires further definition [49].

### Multiple BC cell types

Although a number of the proteins we identified were expressed in just one class of BC cells (ERα-positive, HER2-positive, or TNBC cells), some proteins were overexpressed in more than one BC cell type.

FASN (fatty acid synthase) was highly expressed by MCF-7 (107 SCs) and SK-BR-3 (393 SCs) cells and is also more highly expressed in ERα-positive and HER2-positive mammary tumors (Fig. 1c). FASN catalyzes the synthesis of fatty acids that are required for membrane production in rapidly dividing cells and has been designated a cancer biomarker [50]. Interestingly, while overexpression of FASN has been linked to aberrant cellular architecture, reducing expression of this protein restores nearly normal cellular architecture [51].

PA2G4 (proliferation-associated protein 2G4) was more highly expressed in ERα-positive MCF-7 (129 SCs) and DT28 TNBC (130 SCs) cells. Elevated PA2G4 expression in mammary tumors is associated with decreased patient survival [52, 53]. In contrast, reduced PA2G4 expression is associated with increased cell proliferation and reduced survival of patients with hepatocellular carcinoma [54].

Two proteins were highly expressed in HER2-positive and TNBC cells. AHNAK (neuroblast differentiation-associated protein AHNAK) was the most highly expressed PM protein identified in our study and was especially high in two of the TNBC cell lines, DT22 (931 SCs) and MDA-MB-231 (1219 SCs) cells. The number of AHNAK SCs in these cells was far greater than detected in MCF-10A cells (103 SCs). ANHAK was also highly expressed in HER2-positive SK-BR-3 cells (426 SCs). It has been suggested that AHNAK functions as a tumor suppressor in TNBC cells by decreasing cell proliferation and tumor invasion [55] and that increased expression of this protein may indicate a more favorable prognosis. Consistent with this idea, decreased AHNAK expression is associated with a poor outcome in melanoma patients [56]. SK-BR-3 and DT22 cells also share high expression of TPP2 (tripeptidyl-peptidase 2; 119 and 150 SCs, respectively), a multi-functional enzyme that controls of ERK1 and ERK2 phosphorylation [57].

The eukaryotic translation elongation factors 1-alpha proteins EEF1A1 and EEF1A2 play critical roles in protein synthesis. Peptides from both elongation factors are highly represented in all of the breast cancer cell types whereas the non-transformed MCF-10A cells expressed substantially lower levels of both EEF1A proteins. The overexpression of EEF1A2 in breast cancer results in activation of PIK-Akt-STAT3 pathways and formation of filopodia, resulting in oncogenesis and metastasis [58]. Elevated expression of these elongation factors has also been noted in human ovarian and lung cancer [58].

## Conclusions

Taken together, our findings suggest that the increased expression of specific proteins in BC cells helps to maintain the structural integrity of cellular proteins (AGR2, TCP1) and cellular architecture (FASN), promote the synthesis and procurement of fatty acids and other substrates required for generation of rapidly dividing cells (ACLY, SLC1A5, FASN, GCN1), enhance tumor cell proliferation (AGR2, ACLY, EEF2, EEF1A1, EEF1A2) and invasion (SUSD2), influence tumor cell migration (BASP, TNC), and maintain intracellular homeostasis (PSMA5, ECPAS). The importance of individual proteins in oncogenesis and tumor progression has been demonstrated by inhibiting the expression of proteins in BC cells. For example, inhibiting expression of ACLY [33] reduces BC cell proliferation and knocking down FASN helps to restore normal mammary cellular architecture [51].

Developing targeted treatments for BC tumors would appear to be a daunting task. However, a recurring theme in our findings was that proteins, which were highly expressed in the BC cell lines we examined, are also highly expressed in other types of cancer. Thus, by characterizing tumors by the proteins they express, rather than the tissue of origin, it may be possible to identify therapeutic targets and treatments that would be useful for a variety of tumors. For example, Trastuzumab, which has been utilized as a first line treatment for HER2-positive BC tumors, has also been used to treat HER2-positive gastric cancer [59]. Likewise, the FASN inhibitor C75 has significant antitumor effects on BC cells as well as prostate and ovarian cancer cells [60]. It should be noted, however, that the effect of a protein or an inhibitor can vary in tumors that originate in different tissues.

The veracity of our findings is supported by the fact that clinical trials are currently underway to test the efficacy of novel inhibitors to limit disease progression. A new FASN inhibitor, TVB-2640, is currently being tested for its effect on mammary, colon, prostate, and lung tumors and leukemia (clinicaltrials.gov). Our studies would predict that this inhibitor would be most effective in HER2-positive BC cells. The effectiveness of radioactively-labeled antibodies to TNC is being examined in patients with BC, glioblastoma, and head and neck tumors (clinicaltrials.gov). In addition to their importance as therapeutic targets, some of the highly expressed proteins we have identified (SUSD2, FASN, AHNAK, EEF1A1, EEF1A2) may be useful as diagnostic biomarkers so that disease progression and recurrence might be followed in patients using liquid biopsies. Finally, and perhaps most importantly, the identification of highly abundant cell surface proteins that are unique to an individual’s cancer could result in personalized immunotherapies that are highly effective in controlling or eradicating deadly metastatic disease.

## Additional file

**Additional file 1.** Table S1. Peptide count, NSAF values, EMPAI values, spectral counts, and % sequence coverage for plasma membrane proteins identified by mass spectrometry (FDR < .01).

## Abbreviations

BC: Breast cancer
EMPAI: Exponentially modified protein abundance index
ERα: Estrogen receptor alpha
HER2: Receptor tyrosine-protein kinase HER2
PBS: Phosphate-buffered saline
NSAF: Normalized spectral abundance factor
PDI: Protein disulfide isomerase
PM: Plasma membrane
PR: Progesterone receptor
SC: Spectral count
TNBC: Triple negative breast cancer
